# Ghrelin accelerates wound healing through GHS-R1a-mediated MAPK-NF-κB/GR signaling pathways in combined radiation and burn injury in rats

**DOI:** 10.1038/srep27499

**Published:** 2016-06-07

**Authors:** Cong Liu, Jiawei Huang, Hong Li, Zhangyou Yang, Yiping Zeng, Jing Liu, Yuhui Hao, Rong Li

**Affiliations:** 1 Institute of Combined Injury, State Key Laboratory of Trauma, Burns and Combined Injury, Chongqing Engineering Research Center for Nanomedicine, College of Preventive Medicine, Third Military Medical University, Chongqing, 400038, China

## Abstract

The therapeutic effect of ghrelin on wound healing was assessed using a rat model of combined radiation and burn injury (CRBI). Rat ghrelin, anti-rat tumor necrosis factor (TNF) α polyclonal antibody (PcAb), or selective antagonists of p38 mitogen-activated protein kinase (MAPK), c-Jun N-terminal kinase (JNK), and growth hormone secretagogue receptor (GHS-R) 1a (SB203580, SP600125, and [D-Lys3]-GHRP-6, respectively), were administered for seven consecutive days. Levels of various signaling molecules were assessed in isolated rat peritoneal macrophages. The results showed that serum ghrelin levels and levels of macrophage glucocorticoid receptor (GR) decreased, while phosphorylation of p38MAPK, JNK, and p65 nuclear factor (NF) κB increased. Ghrelin inhibited the serum induction of proinflammatory mediators, especially TNF-α, and promoted wound healing in a dose-dependent manner. Ghrelin treatment decreased phosphorylation of p38MAPK, JNK, and p65NF-κB, and increased GR levels in the presence of GHS-R1a. SB203580 or co-administration of SB203580 and SP600125 decreased TNF-α level, which may have contributed to the inactivation of p65NF-κB and increase in GR expression, as confirmed by western blotting. In conclusion, ghrelin enhances wound recovery in CRBI rats, possibly by decreasing the induction of TNF-α or other proinflammatory mediators that are involved in the regulation of GHS-R1a-mediated MAPK-NF-κB/GR signaling pathways.

Combined radiation and burn injury (CRBI) is a classical type of combined radiation injury (CRI), where a major radiation injury is accompanied by burn, simultaneously or consecutively^1^. CRBI usually occurs after a nuclear accident and may severely threaten human health without proper intervention^2^,^3^. CRBI is much more complex and difficult to treat than a single injury (radiation or burn), with a higher risk of early shock, more severe suppression of hematopoietic and immunologic functions, extensive gastrointestinal damage, and delayed wound healing^1^,^4^,^5^. However, the lack of clinical cases restricts CRBI research, which necessitates the use of CRBI animal models^6^,^7^,^8^.

Acute severe inflammatory response (ASIR) induced by endogenous gastrointestinal or/and respiratory tract infection, and exogenous infection caused by impaired wound healing, is an important cause of death of CRBI animals^1^,^9^,^10^. Conversely, ASIR may delay wound regeneration, thus worsening CRBI symptoms^11^. Bacteria from impaired burn wounds were detected in increasing amounts in the liver and the circulation as CRBI progressed, aggravating the inflammatory response^1^. Radiation or burn injury can each cause systemic inflammation^12^.

Immune cells are a major source of most proinflammatory mediators, such as tumor necrosis factor (TNF) α, interleukin (IL) 6, and IL-1β. The immune cells, especially macrophages, are distributed in the body, but after radiation or/and activation by primary proinflammatory mediators^13^,^14^, they accumulate at CRBI wound sites and produce cytokines that can influence the wound healing progress^15^,^16^. The common inflammatory cytokine TNF-α is necessary for the initiation of wound healing process^17^. However, TNF-α inhibits wound healing when overexpressed, e.g., during sepsis or severe CRBI^13^,^18^. Blocking TNF-α overexpression enhances wound healing^19^,^20^. The expression of TNF-α predominantly depends on the activation of mitogen activated protein kinase (MAPK) p38, c-Jun N-terminal kinase (JNK), and extracellular signal-regulated kinase (ERK) classical signaling pathways (collectively known as the MAPK signaling pathways), as well as the nuclear factor (NF) κB pathway^21^.

Acute stress response (ASR) takes place in the early stage of CRBI and is mostly attributed to excessive activation of the hypothalamic pituitary adrenal (HPA) axis^22^. During ASR, adrenal gland glucocorticoid (GC) serum levels rise slightly. GCs interact with a cytoplasmic glucocorticoid receptor (GR)^23^. Activated, usually phosphorylated, GC-GR protein dimers translocate into the nucleus and bind specific DNA sequences called glucocorticoid response elements (GREs). This results in diverse events, such as the widely known anti-inflammatory effect^24^,^25^. However, in severely burned subjects, both humans and animals, GC levels markedly increase whereas GR expression decreases, which leads to glucocorticoid resistance (GCR)^26^,^27^. GCR weakens the anti-inflammatory effect of GC.

Ghrelin is a recently discovered multifunctional gastrointestinal peptide hormone involved in various biological processes. It interacts with its endogenous growth hormone secretagogue receptor (GHS-R) 1a^28^. Ghrelin levels decreased in irradiated rats and exogenous human ghrelin administration improved animal survival^29^. Ghrelin also alleviated organ injury and improved survival of irradiated rats with severe sepsis, by weakening inflammatory responses^30^,^31^. It has been reported that ghrelin helps to alleviate CRBI symptoms^32^; however, detailed mechanisms of ghrelin-accelerated CRBI wound healing remain largely unknown.

This study was performed to verify the wound healing effect of ghrelin in CRBI rats, exploring the possible molecular mechanisms involving GHS-R1a, inflammatory signaling, and TNF-α. MAPK and GR signaling pathways interact, and we also hypothesized that the inhibition of MAPK signaling pathways may attenuate GCR, possibly contributing to ASIR mitigation, wound recovery, and a good CRBI outcome.

## Results

### CRBI Treatment Results In Decrease Of Serum Ghrelin Levels

**Figure 1:**
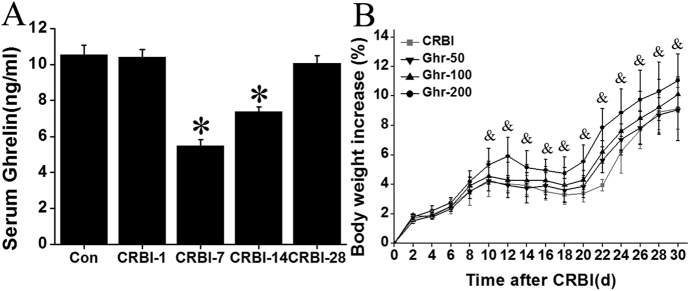
Determination of ghrelin levels and body weight change. (**A**) Alterations in ghrelin serum levels of uninjured rats (Con) or CRBI rats, at days 1 (CRBI-1), 7 (CRBI-7), 14 (CRBI-14), and 28 (CRBI-28) after injury. (**B**) Body weight increase (%) of CRBI rats treated with saline, 50 nmol/kg (Ghr-50), 100 nmol/kg (Ghr-100), or 200 nmol/kg (Ghr-200) ghrelin, over 30 day period. Body weights were recorded every 2 days. Data are presented as means ± SE (n = 5–7) and were analyzed by one-way ANOVA and Student-Newman-Keuls test: *P < 0.05 versus control group, ^&^*P* < 0.05 versus CRBI group.

Serum ghrelin levels in untreated rats were about 10 ng/mL (Fig. 1A). The serum ghrelin levels gradually decreased after CRBI, reaching the lowest value (ca. 5 ng/mL) at day 7 (Fig. 1A), and then slowly increased until they reached the initial concentration at day 28. Ghrelin levels in CRBI rats were statistically lower than in untreated rats (*P* < 0.05; Fig. 1A) for about a 2-week period.

### Ghrelin Treatment Improves CRBI Rat Weight Gain In A Dose-Dependent Manner

The body weights of ghrelin-treated and -untreated CRBI rats increased for about 10 days in the initial stage of injury (Fig. 1B). The weight of animals receiving 200 nmol/kg ghrelin showed the greatest increase, by 5% (~11 g); this increase was significant compared to ghrelin untreated CRBI rats (*P* < 0.05). Weights of all ghrelin-supplemented animals increased until day 10, when they leveled off, and remained constant until day 18. During this period, the CRBI rats not treated with ghrelin suffered an obvious weight loss. Interestingly, about 20 days later, their weight increased again (9–11% at day 30), which was further sustained (Fig. 1B). Body weights of CRBI rats receiving different doses of ghrelin increased in a dose-dependent manner (Fig. 1B).

During this process of body weight evaluation, the hematopoietic function of CRBI rats treated with or without ghrelin was also evaluated at 3, 7 and 14 days after injury, by measuring the count of RBC, WBC and PLT in the peripheral blood. This result can be found in supplementary information (Fig. S1).

### Exogenous Ghrelin Administration Accelerates Wound Healing In CRBI Rats

**Figure 2:**
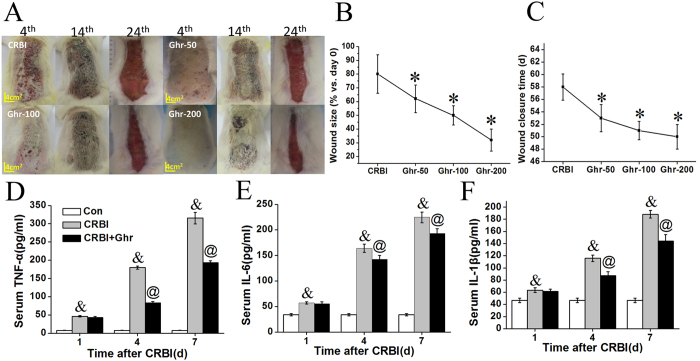
Wound healing and anti-inflammatory effects of ghrelin on CRBI rats. (**A**) Macroscopic observation of wound healing on days 4, 14, and 24 after CRBI. CRBI rats were treated with normal saline or various doses of ghrelin (50, 100 or 200 nmol/kg) (Ghr-50, Ghr-100, Ghr-200) in normal saline, continuously, for 7 days. (**B**) Comparison of wound sizes (%) of CRBI rats with or without ghrelin treatment 24 days after CRBI. (**C**) Wound closure time. (**D**) Serum levels of TNF-α, (**E**) IL-6, and (**F**) IL-1β in uninjured rats (Con) or CRBI rats, treated with normal saline (CRBI) or 200 nmol/kg ghrelin (CRBI+Ghr). Representative results are given, and cytokine concentrations were measured by ELISA. Data are presented as means ± SE (n = 5–7) and were analyzed by one-way ANOVA and Student-Newman-Keuls test: *P < 0.05 versus CRBI group; ^&^*P* < 0.05 versus control group; ^@^*P* < 0.05 versus CRBI group on days 4, 7 after injury, respectively.

Wound healing data were collected and analyzed at 4, 14, and 24 days post CRBI, with respect to wound healing status (Fig. 2A), wound size (presented as percent of the affected area on day 0) at day 24 (Fig. 2B), and wound closure times (Fig. 2C). Severe subcutaneous hemorrhage occurred and different numbers and sizes of hemorrhagic spots were observed in the early healing stage (i.e., 4 days post injury). Ghrelin treatment decreased hemorrhagic spot counts, in a dose-dependent manner (Fig. 2A). Injured wounds were covered with crusts and no significant differences were observed between groups in the mid-healing stage (i.e., 14 days post injury) (Fig. 2A); the wound healing process was initiated during this period. After the crusts detached in the late healing stage (i.e., 24 days post injury), the contracted wounds enabled measurements of wound sizes. Wound sizes of CRBI rats receiving ghrelin treatment were smaller than those in CRBI rats treated with saline (Fig. 2A). At day 24, the average remaining wound areas of saline-treated CRBI rats, and rats receiving 50 nmol/kg, 100 nmol/kg, or 200 nmol/kg ghrelin were 40 cm^2^ (~80%), 31 cm^2^ (~60%), 25 cm^2^ (~50%), and 16 cm^2^ (~30%), respectively (Fig. 2B). Ghrelin significantly promoted wound healing of CRBI rats in a dose-dependent manner (*P* < 0.05; Fig. 2B). The wounds of saline-treated CRBI rats closed 50–60 days after CRBI, while those of ghrelin-treated CRBI rats healed, on average, 3–8 days earlier, and the difference was significant (*P* < 0.05; Fig. 2C) compared to saline-treat CRBI rats.

### Ghrelin Inhibits The Induction of Proinflammatory Cytokines In CRBI Rats

As shown in Fig. 2D, serum levels of TNF-α increased more than 6-fold on day 1 after CRBI, and continued to increase more than 22- and 41-fold at days 4 and 7 after injury, respectively, compared to those in normal rats. Ghrelin treatment led to significantly decreased serum levels of TNF-α, by 54% on day 4, and 39% on day 7, post CRBI (*P* < 0.05). Serum levels of IL-6 increased about 1.7-, 4.8-, and 6.6-fold, on days 1, 4, and 7 after CRBI, respectively (Fig. 2E). Ghrelin treatment led to significantly decreased serum levels of IL-6, by 13% on day 4 and 14% on day 7 after CRBI (*P* < 0.05; Fig. 2E). Similarly, serum levels of IL-1β increased by about 1.4-, 2.5-, and 4.0-fold, on days 1, 4, and 7 after CRBI, respectively (Fig. 2F). Notably, ghrelin treatment led to significantly decreased serum levels of IL-1β, by 25% on day 4 and 23% on day 7 post CRBI (*P* < 0.05; Fig. 2F). To summarize, ghrelin decreased the induction of TNF-α, IL-6, and IL-1β in CRBI rats, especially the TNF-α.

### CRBI Enhances Phosphorylation Of Signaling Molecules And Results In Decreased Glucocorticoid Receptor Levels

**Figure 3:**
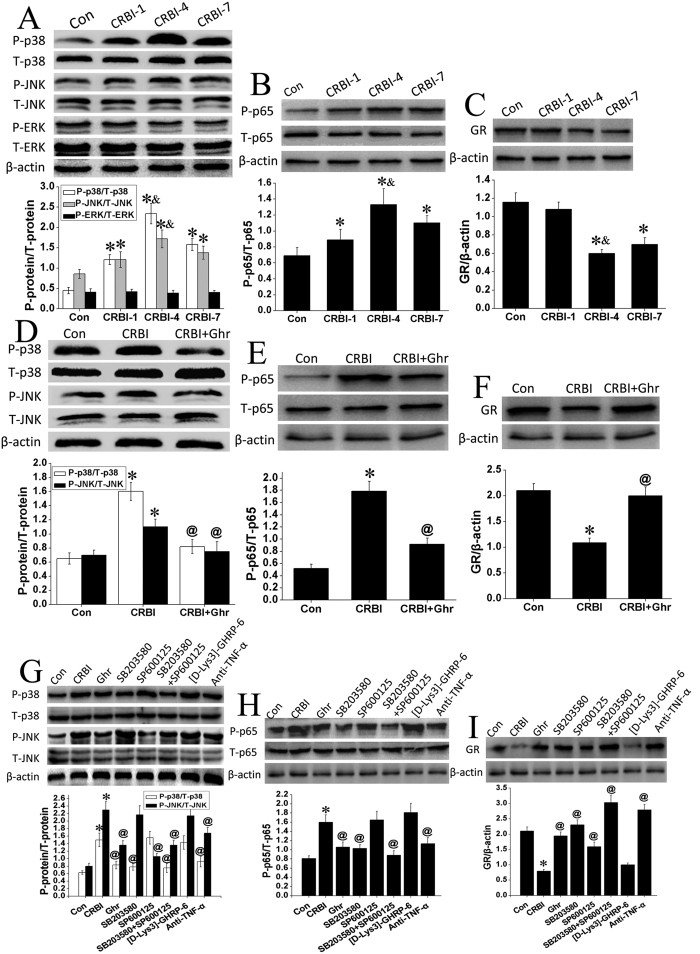
Effects of ghrelin on signaling molecular levels in rat peritoneal macrophages. (**A**) Phosphorylation and overall protein levels of p38MAPK, JNK, ERK, (**B**) p65 NF-κB, and (**C**) GR proteins in peritoneal macrophages from uninjured rats (Con) and CRBI rats at days 1 (CRBI-1), 4 (CRBI-4), and 7 (CRBI-7) after injury. (**D**) Effects of ghrelin on P-p38MAPK, P-JNK, (**E**) P-p65 NF-κB, and (**F**) GR levels in uninjured rats (Con) or CRBI rats treated with normal saline (CRBI) or 200 nmol/kg ghrelin (CRBI + Ghr), at 4 days after injury. (**G**) Alterations of P-p38MAPK, P-JNK, (**H**) P-p65 NF-κB, and (**I**) GR levels in uninjured rats (Con), and CRBI rats treated with either normal saline (CRBI), 200 nmol/kg ghrelin (Ghr), SB203580, SP600125, co-administered SB203580 and SP600125, [D-Lys3]-GHRP-6 (given before each ghrelin administration), or anti-TNF-α antibody, at 4 days after injury. These inhibitors were administered subcutaneously or intravenously for seven consecutive days. The protein levels were detected as described in *Methods* section. β-actin, as a loading control, was detected at the same gel. All gels from each experiment were run under the same experimental conditions, and the blots were cropped and grouped together into a single figure. Representative results are given and histograms are drawn based on the gray value of each band calculated by Image J software. Data are presented as means ± SE (n = 3–5) and were analyzed by one-way ANOVA and Student-Newman-Keuls test: *P < 0.05 versus control group; ^&^*P* < 0.05 versus corresponding CRBI-1 or CRBI-7 group; ^@^*P* < 0.05 versus corresponding CRBI (day 4) group.

Specific protein (total or phosphorylated, i.e., p38 MAPK or P-p38 MAPK) levels from isolated peritoneal macrophages were detected by western blotting. Compared to findings for uninjured controls, the phosphorylated fraction of p38 MAPK (P-p38MAPK) increased 2.7-, 5.3-, and 3.5- fold, on days 1, 4, and 7, respectively, after CRBI (*P* < 0.05; Fig. 3A). Similarly, phosphorylation of JNK (P-JNK) increased 1.4-, 2.0-, and 1.6-fold, on days 1, 4, and 7 post CRBI, respectively (*P* < 0.05; Fig. 3A). No obvious alteration in phosphorylation was observed for ERK (Fig. 3A). Phosphorylation of p65 NF-κB increased 1.3-, 1.9-, and 1.6-fold, compared to the untreated control group, on days 1, 4, and 7 after CRBI, respectively (*P* < 0.05; Fig. 3B). In comparison, the overall levels of GR significantly decreased, by 48% on day 4, and 40% on day 7, after CRBI, correspondingly (*P* < 0.05; Fig. 3C). These results implied that these intracellular signaling molecules play an important role in the development of inflammatory response in CRBI rats. Day 4 was selected as the optimal time point to assess the effects of ghrelin on these signaling molecules.

### Ghrelin Inhibits Phosphorylation Of Signaling Molecules And Leads To Increased Glucocorticoid Receptor Levels After CRBI

CRBI resulted in increased phosphorylation p38 MAPK, JNK, and p65 NF-κB by about 2.5, 1.5 and 3.4 fold at day 4, respectively, compared to saline-injected CRBI rats (*P* < 0.05; Fig. 3D,E). Ghrelin treatment (200 nmol/kg) significantly decreased P-p38MAPK, P-JNK, and P-p65 NF-κB levels at day 4, by 49%, 32%, and 49%, respectively (*P* < 0.05; Fig. 3D,E). The overall levels of GR on day 4 post CRBI increased 1.8-fold after such ghrelin treatment, compared with saline-CRBI group (P < 0.05; Fig. 3F).

The function of GR was partly regulated by HPA axis and the GHS-R1a was highly expressed in hypothalamus, therefore we observed the expression levels of GHS-R1a in hypothalamus of CRBI rats with or without ghrelin treatment on day 4. The detailed result was available in supplementary information (Fig. S2).

### Effects Of Signaling Pathway Inhibitors On Signaling Molecule Phosphorylation And Glucocorticoid Receptor Levels

Specific inhibitors were used to verify that the effects of ghrelin on the management of inflammatory response and wound healing were indeed mediated through inhibition of p38 MAPK- and JNK- signaling pathways in a GHS-R1a-dependent manner. The following signaling blockers were used: SB203580, for p38 MAPK; SP600125, for JNK; and [D-Lys3]-GHRP-6 for GHS-R1a. Four days after CRBI, the phosphorylation levels of p38 MAPK and JNK increased 2.4- and 2.8-fold, respectively (*P* < 0.05; Fig. 3G), while p65 NF-κB increased 2.0-fold (*P* < 0.05; Fig. 3H) compared with uninjured control group. The levels of these proteins decreased by 45%, 39%, and 34%, for p38 MAPK, JNK, and p65 NF-κB, respectively, after 4 days of continuous ghrelin (200 nmol/kg) administration (*P* < 0.05; Fig. 3G,H). When administered separately, SB203580 and SP600125 inhibited the phosphorylation of p38MAPK and JNK by 48% and 54%, respectively (*P* < 0.05; Fig. 3G) and SB203580 also significantly inhibited the phosphorylation of p65 NF-κB by 36% (*P* < 0.05; Fig. 3H). Co-administration of these two blockers markedly decreased the phosphorylation of p38MAPK, JNK, and p65 NF-κB, by 50%, 41%, and 45%, respectively, compared with saline-CRBI group (P < 0.05; Fig. 3G,H). However, SB203580 did not decrease protein levels of JNK, while SP600125 did not decrease levels of p38 MAPK (Fig. 3G). Administration of [D-Lys3]-GHRP-6 before ghrelin treatment inhibited the ghrelin-mediated decrease of P-p38MAPK, P-JNK, and P-p65 NF-κB; no obvious changes were observed, compared with saline treated CRBI group (Fig. 3G,H). Anti-TNF-α antibody administration decreased the abundance of P-p38MAPK, P-JNK, and P-p65 NF-κB by 39%, 27%, and 29%, respectively (*P* < 0.05; Fig. 3G,H).

As described above, ghrelin administration resulted in elevated levels of GR in CRBI rats. SB203580 and SP600125 increased GR levels 2.9- and 2.0-fold, respectively, and blocker co-administration increased GR levels 3.8-fold (*P* < 0.05; Fig. 3I). Use of [D-Lys3]-GHRP-6 before ghrelin treatment failed to increase GR levels, while anti-TNF-α antibody increased them 3.5-fold (*P* < 0.05; Fig. 3I).

### The Effect Of Signaling Pathway Inhibitors On Serum TNF-α Levels

**Figure 4:**
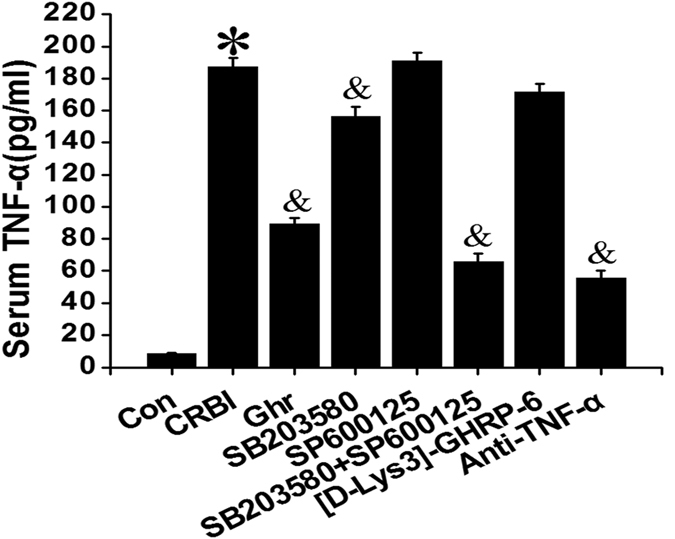
Serum levels of TNF-α in uninjured rats (Con), or CRBI rats treated with normal saline (CRBI), 200 nmol/kg ghrelin (Ghr), SB203580, SP600125, co-administered SB203580 and SP600125, [D-Lys3]-GHRP-6 (given before each ghrelin administration), or anti-TNF-α antibody, at 4 days after injury. These inhibitors were administered subcutaneously or intravenously for seven consecutive days. Concentrations of TNF-α were measured by ELISA. Data are presented as means ± SE (n = 5–7) and were analyzed by one-way ANOVA and Student-Newman-Keuls test: *P < 0.05 versus control group; ^&^*P* < 0.05 versus CRBI group.

Four days after CRBI, serum TNF-α levels significantly increased by approximately 20-fold (from 9 pg/mL to 180 pg/mL), compared to those for the uninjured control (*P* < 0.05; Fig. 4). Administration of ghrelin, SB203580, anti-TNF-α antibody, or a combined injection of SB203580 and SP600125 significantly decreased TNF-α levels by 52%, 17%, 70%, and 65%, respectively (*P* < 0.05). Administration of the JNK inhibitor SP600125 had no apparent effect on TNF-α levels (Fig. 4). Administration of [D-Lys3]-GHRP-6 before ghrelin dosing (200 nmol/kg) did not alter serum TNF-α levels in CRBI rats (Fig. 4).

Otherwise, we investigated the anti-inflammation effect of ghrelin in LPS treated peritoneal macrophages isolated from CRBI rats. This result can be found in supplementary information (Fig. S3).

### The Effect Of Signaling Pathway Inhibitors On Wound Healing In CRBI Rats

**Figure 5:**
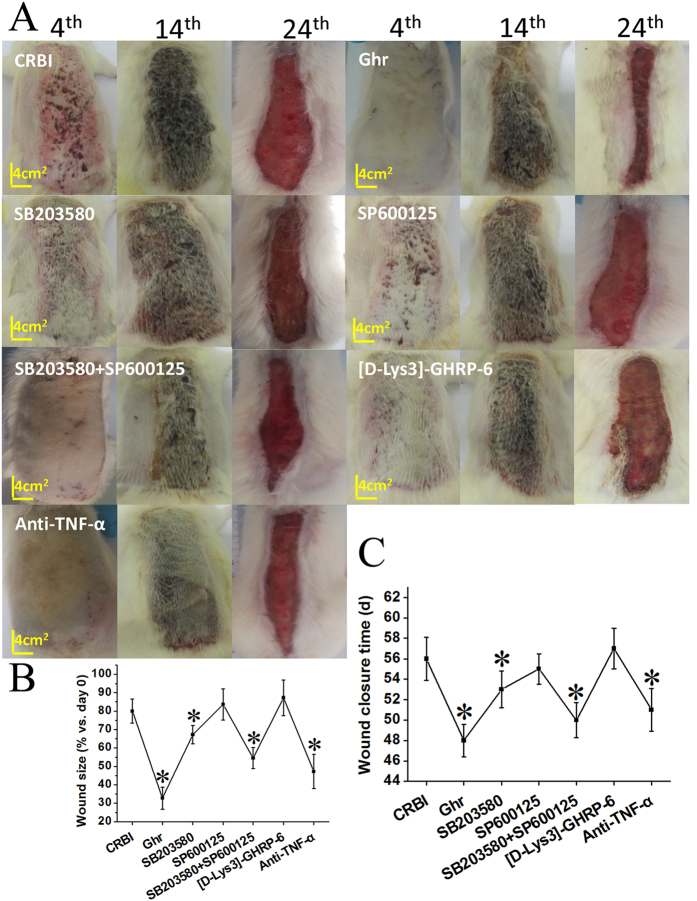
Effects of ghrelin and other inhibitors on wound healing in CRBI rats on days 4, 14, and 24 after injury. (**A**) Macroscopic observation of wound healing conditions on days 4, 14, and 24 after CRBI. CRBI rats were treated with normal saline (CRBI), ghrelin (Ghr), SB203580, SP600125, co-administered SB203580 and SP600125, [D-Lys3]-GHRP-6 (given before each ghrelin administration), or anti-TNF-α antibody. (**B**) Comparison of wound sizes (%) of CRBI rats treated with ghrelin or inhibitors 24 days after CRBI. (**C**) Wound closure time. Representative results are given. Data are presented as means ± SE (n = 5−7) and were analyzed by one-way ANOVA and Student-Newman-Keuls test: *P < 0.05 versus CRBI group.

In the initial stage of CRBI, bleeding spot counts increased gradually and quickly spread over the injured wounds. After the administration of ghrelin, co-injection of SB203580 with SP600125, or the anti TNF-α antibody, wounds improved markedly (Fig. 5A). In the middle stage of CRBI, the scabs formed and tended to be hard; no macroscopic differences were observed between groups. However, in the late stage of healing, the detachment of scabs enabled the determination of wound size. As shown in Fig. 5A,B, the average remaining unhealed wound area was about 42 cm^2^ at 24 days after CRBI. The administration of ghrelin or anti- TNF-α antibody, or co-administration of SB203580 with SP600125, significantly accelerated the closure of injured wounds by approximately 47%, 33%, and 26%, respectively (*P* < 0.05). Administration of SB203580 had a relatively weak wound healing effect (13%) compared with other groups. However, application of [D-Lys3]-GHRP-6 almost completely inhibited the positive effect of ghrelin on wound healing (Fig. 5B). Advanced wound closure was observed in groups treated with ghrelin, SB203580 together with SP600125, and anti TNF-α antibody for about 8, 5, and 5 days, respectively (*P* < 0.05; Fig. 5C).

The wound healing effect of ghrelin on CRBI rats seemed to be realized by inhibiting systemic inflammation. To explore the possible local effect of ghrelin on wound healing, we determined the expression levels of GHS-R1a in the skin during wound healing (Fig. S4). We observed that most epidermal cells in wound tissues from each group didn’t express GHS-R1a (Fig. S4A). However, most cells especially the fibroblasts distributed in the dermis (granulations) expressed GHS-R1a significantly (Fig. S4). Ghrelin treatment markedly improved the decreased GHS-R1a expression in CRBI rats (Fig. S4B).

## Discussion

Ghrelin is an endogenous “brain-gut” peptide that participates in various biological processes through interacting with its specified receptor GHS-R1a located in cell membranes^33^. As a G-protein-coupled receptor, GHS-R1a is distributed in the body, and thus ghrelin exerts multiple biological functions, including the widely acknowledged anti-inflammatory property^28^,^33^,^34^. CRBI causes cardiovascular system dysfunction, gastrointestinal damage and immunosuppression, and ghrelin might theoretically be involved as a potent hormone to attenuate these injuries^29^,^34^,^35^. However, myelo-suppression and acute systemic inflammatory response are the mainly recognized causes of early death of CRBI animals^1^. In this study, we provide evidence that ghrelin mitigates the systemic inflammatory response and thus improves wound healing, which may contribute to the reduced mortality of CRBI rats.

The wound healing processes in CRBI rats undergoes three overlapping but distinct stages: inflammation, new tissue formation, and remodeling^36^. Inflammation occurs immediately after tissue damage, and neutrophils and macrophages are the major cell types that appear in the wounds 2–3 days after injury. However, in this early stage, radiation exposure destroys the hematopoiesis and induces a dramatic decrease of platelets (PLT) and white blood cells (WBC), especially neutrophils and lymphocytes^37^. Large amounts of proinflammatory cytokines, mainly secreted by activated macrophages, appear in circulation and flood the wounds, suppressing hematopoiesis and interfering with wound healing^38^. This stage lasts for a relatively long time (about 7 days), and delays new tissue formation and remodeling. New tissue formation is also accompanied by excessive inflammation until apoptosis of most macrophages and removal of most devitalized tissues^36^; therefore, wound closure time is greatly delayed. In CRBI rats, the increased skin vaso-permeability enables ghrelin penetration into injured tissues and protects skin from further damage, in agreement with the results of our study. It was previously shown that ghrelin decreases microvascular leak during inflammation through the GHS-R1a mediated NF-κB pathway^39^. The suppression of hematopoietic function is one of the causes of delayed wound healing in CRBI rats, and our results showed a decrease of WBC and PLT after CRBI but an obvious increase after ghrelin treatment in CRBI rats on days 3, 7, and 14 after injury (see Supplementary Fig. S1). Our results also showed that ghrelin decreased serum TNF-α, IL-6, and IL-1β levels in CRBI rats on days 1, 4, and 7 after injury. These indicated that ghrelin inhibits the excessive inflammatory response, enhances the recovery of hematopoietic function, and maintains the biological balance between inflammation and hematopoiesis^38^,^40^,^41^.

Whether ghrelin has a direct relationship with hematopoiesis needs further investigation. MAPK and NF-κB signaling pathways are widely studied^21^,^42^. In CRBI rats, the serum levels or phosphorylation of p38 MAPK and JNK significantly increased, while ERK remained constant and NF-κB was activated. This finding was similar to that of other studies with inflammatory models. In addition, we found that GR was down-regulated after CRBI, which may be responsible for the occurrence of GCR, leading to decreased anti-inflammatory effects^27^. Ghrelin administration inhibited the phosphorylation of p38 MAPK, JNK, and NF-κB, and increased GR levels. Selective blocking of either p38 MAPK or JNK had a smaller effect on increasing GR levels than co-administration of these two blockers. This indicated that a negative regulation may exist between the MAPK pathway and GR in peritoneal macrophages of CRBI rats. Previously, the role of MAPK in regulating GC-GR function was investigated in burn injury in mice overexpressing p38 MAPK, which resulted in reduced GC functions^43^. It has been also described that the inhibition of p38 MAPK or JNK pathway increased the GRα/GRβ ratio (GRα is the active form of GR) and GC affinity, thus improving the GC function^44^. However, based on our data, p38 MAPK seems to play a rather important role in the regulation of GC or GR functions. TNF-α is an important proinflammatory cytokine influencing wound healing, and can be utilized as a therapeutic target during impaired wound healing, including burn injury^19^,^45^,^46^. Our study revealed that the administration of anti-rat TNF-α antibody inhibited the activation of p38 MAPK, JNK, and NF-κB pathways, increased the levels of GR, and, finally, enhanced wound recovery in CRBI rats. As with the administration of each blocker of the MAPK pathways, serum TNF-α levels decreased correspondingly, and to different extents, and this implied that ghrelin may improve wound healing in CRBI rats by simply decreasing the induction of TNF-α. In addition, when [D-Lys3]-GHRP-6 was used to block GHS-R1a, ghrelin lost its anti-inflammatory property. That was followed by a delayed wound healing, thus confirming that the wound healing effect of ghrelin in CRBI rats was GHS-R1a dependent.

As has been stated, ghrelin levels in CRBI rats decreased. However, the expression levels of GHS-R1a in tissues were not studied. Using immunohistochemistry (IHC), we occasionally observed decreased levels of GHS-R1a in hypothalamus of CRBI rats, which returned to normal after ghrelin administration (see Supplementary Fig. S2). We previously discussed that over-activation of HPA may have led to GR decrease and GCR. Thus, GR increase in CRBI rats after ghrelin treatment was possibly associated with the inhibition of HPA through up-regulation of GHS-R1a in hypothalamus^47^. Therefore, the anti-inflammatory and wound healing properties of ghrelin were possibly strongly associated with up-regulated GHS-R1a levels. It has been established that the stimulation of the vagus nerve inhibits the inflammatory response in burned mice, and also blocks the release of proinflammatory cytokines (TNF-α, IL-6, and IL-1β) from lipopolysaccharide-induced peritoneal macrophages (LPS)^48^. In a rat model of sepsis, ghrelin was thought to suppress inflammation indirectly, by inactivating the sympathetic nerve and, thus, activating the vagus nerve^49^,^50^, involved in the regulation of ‘cholinergic anti-inflammatory pathway’^51^. Additionally, cervical sympathetic block (CSB) was previously used as an effective measure to treat CRBI in our institute^1^. CSB significantly improved the survival of CRBI mice mostly by decreasing the overexpression of TNF-α, IL-6, and IL-1β. Given these, we speculate that the anti-inflammation property of ghrelin was also partly achieved by regulating the excitatory balance between the sympathetic nerve and vagus nerve. Ghrelin was identified as non-effective in directly decreasing the proinflammatory cytokines secreted by peritoneal macrophages stimulated by LPS^50^. However, studies also showed that ghrelin decreased the proinflammatory cytokines produced by RAW264.7 macrophages treated with LPS in a dose- and time- dependent manner^52^. Our previous study also showed that the levels of proinflammatory mediators in supernatants of cultured peritoneal LPS-induced macrophages decreased after treatment with different doses of ghrelin (see Supplementary Fig. S3). Therefore, we suspect that ghrelin suppresses inflammation through activation of vagus nerve and direct interaction with peritoneal macrophages, namely, through neurohumoral regulation (see Supplementary Fig. S5).

Additionally, we preliminary observed the local effects of ghrelin in wound healing, by determining the expression levels of GHS-R1a in the skin (granulation tissues) of CRBI rats with or without ghrelin treatment. As shown in Supplementary Fig. S4, GHS-R1a was rarely distributed in the epidermis, whereas highly expressed in the dermis of granulation tissues, especially the fibroblasts. CRBI resulted in decreased fibroblast number and GHS-R1a expression. Ghrelin treatment increased the fibroblast number and GHS-R1a expression, thus partly promoting the synthesis and secretion of collagen and certain growth factors, leading to improved wound healing. In conclusion, it is more reliable that ghrelin accelerates wound healing through GHS-R1a-mediated systemic and local effects in CRBI rats.

Herein, we performed an observational study of wound healing in the CRBI rat model. The role of vagus verve in regulating wound healing of CRBI rats deserves further studies. The direct mechanisms and effects of ghrelin on the repair and regeneration of wounds of CRBI rats also need further investigation. We here show that ghrelin can enhance wound healing in CRBI rats, but whether it would have a similar effect in clinical trials remains unknown. However, the increased experience with ghrelin administration in human beings indicates that ghrelin possesses the potential to become an extended therapeutic agent in various patient populations^53^.

## Methods

### Animals

Healthy adult male Sprague-Dawley rats (age: 6–8 weeks; body weight: 180–200 g) were purchased from the experimental animals center of the Third Military Medical University (TMMU). The rats were housed in a temperature- and humidity-controlled specific pathogen free room, with a 12-h light/dark cycle, reared on a standard Purina rat chow diet. After purchasing, the animals were housed separately in plastic boxes and were allowed to acclimatize for 7 days. All animal experimental procedures were performed in accordance with the Guide and Use of Laboratory Animals (Institute of Laboratory Animal Resources). The Institutional Animal Care and Use Committee for Medical Research at TMMU approved this study.

### Rat CRBI Model

All animals were fasted overnight before sequential radiation and burn (thermal) injuries. The rats were individually housed in specially designed rotating plastic cylinders with pores. The animals received 5 Gy radiation (γ-ray radiation from a Cobalt-60 source [China Institute of Atomic Energy, Beijing, China.]), at approximately 0.70 Gy/min, as controlled by a γ-ray dose monitor (Automess, Germany). The rats were then allowed food and water *ad libitum*. Thermal injury was inflicted within 1 h after radiation. The rats were anesthetized with 1% pentobarbital sodium (50 mg/kg body weight [BW]) intraperitoneal (i.p.) injection, individually fixed face down to a wooden board, and shaved with an electric clipper. The board with the fixed animal was placed 70 cm underneath a 5 kW bromine-tungsten lamp (GuangYao Inc., Shanghai, China). Fifteen percent of the total body surface area (TBSA) calculated according to the Meeh-Rubner formula (TBSA[m^2^] = 0.0913 × {BW[kg]}^2/3^) was marked out. Asbestos clothes protected half the skin area, while the other half was exposed (28 s), following which the treatment was reversed, which warrantied reproducible thermal injury^1^. Immediately after the injury, sterilized normal saline (40 mL/kg BW) was i.p. injected to prevent early shock.

### Ghrelin Administration

Immediately after CRBI, rat ghrelin (Chinese Peptide Company, Hang Zhou, China) was administered by continuous subcutaneous infusion using an 200-μL mini-osmotic pump (Alzet, DURECT corporation in Canada, infusion rate 8μL/h) for 7 days, with the pump reloaded every 24 h. Ghrelin doses of 50 nmol · kg^−1^ · d^−1^, 100 nmol · kg^−1^ · d^−1^, and 200 nmol · kg^−1^ · d^−1^ were chosen based on our experience and literature data^31^,^54^. CRBI control rats received equivalent volumes (~200μL) of sterilized normal saline.

### Measurement Of Serum Ghrelin Levels

Blood samples (n = 5–7/group) were collected into sterilized Eppendorf(EP) tubes at 1, 7, 14, and 28 days after CRBI, and centrifuged (1,500 × *g*, 15 min, at 4 °C). The supernatants were immediately stored at −80 °C until further experiments. Enzyme Immunoassay Kit (Phoenix Pharmaceuticals, Inc., Burlingame, CA) was used to determine the serum ghrelin levels according to the manufacturer’s instructions. Ghrelin concentrations were calculated based on the standard curve.

### Weight Change Evaluation

Ghrelin is as growth hormone (GH)-releasing peptide^33^. Therefore, weight change was chosen as an indicator of the effect of ghrelin treatment on CRBI rats (n = 5–7/group). Designated animal breeder recorded the starting animal weights on the day of CRBI (day 0). The weights were recorded every 2 days for 30 days, and the percent increase of body weight was presented at various time points.

### Wound Healing Evaluation

Wound healing was evaluated visually, by taking photos, every 2 days. The progress of wound healing was compared at 4, 14, and 24 days after CRBI, based on the number of hemorrhagic necrotic spots, skin wound size, and wound closure times. For the purpose of this study, preservative film was attached to the wound surface on day 24, and the wound outlined with a marking pen. Wound size (cm^2^) was calculated indirectly through the film and presented as a percent of the affected area on day 0. Wound closure time was determined based on the degree of epithelization.

### Cytokine Determination

Blood samples (n = 5–7/group) from normal(untreated) rats and CRBI rats receiving saline or 200 nmol · kg^−1^ · d^−1^ ghrelin were collected separately into sterilized EP tubes at 1, 4, and 7 days after CRBI. They were centrifuged (1,500 × *g*, 15 min, at 4 °C), and serum samples were harvested, and stored at −80 °C until assayed. Cytokines were measured via enzyme-linked immune sorbent assay (ELISA) using Enzyme Immunoassay Kit (Sangon Biotech, China), according to manufacturer’s instructions.

### Peritoneal Macrophage Isolation

Rats were anesthetized by intravenous (i.v.) administration of pentobarbital sodium (50 mg/kg BW). Rat peritoneal macrophages were harvested by sterile lavage of the peritoneal cavity with 20 mL cold RPMI medium (without fetal bovine serum and antibiotics, Gibco, USA). After 10 min, the lavage fluid was collected and centrifuged (1,200 × *g*, 6 min). Supernatant was removed and red blood cell (RBC) lysis buffer (Beyotime, China) was added. After 3 min, the cell suspension was centrifuged (1,200 × *g*, 3 min), the supernatant decanted, and the cells washed with cold phosphate buffered saline (PBS, pH 7.4). The cells were then resuspended in RPMI 1640 supplemented with 10% FBS, 100 ng/mL penicillin, and 100 ng/mL streptomycin, and incubated at 37 °C for 2 h. Nonadherent cells were then removed by washing with PBS. The adherent macrophages were used for protein extraction.

### Western Blot Analysis

Isolated peritoneal macrophages were collected using a cell scraper and homogenized in lysis buffer (50 mM Tris, 150 mM NaCl, 1% NP-40, 0.5% sodium deoxycholate, 0.1% SDS, 1 mM EDTA, 1 mM PMSF) for 15 min on ice. Supernatants were collected after centrifugation (14,000 × *g*, 10 min, at 4 °C) and used immediately or stored at −80 °C until assaying. Protein concentrations were determined using BCA Protein Assay Kit (Beyotime, China), and were adjusted to 3–5 mg/mL. Protein samples containing 20% loading buffer were denatured at 99 °C for 10 min. After cooling to room temperature, the samples were loaded on 5–12% Tris-glycine SDS polyacrylamide gels, electrophoresis (SDS-PAGE) was conducted at 90 V, and the resolved proteins transferred to a 0.45 μm polyvinylidene fluoride (PVDF) membrane (90 V for 110 min). PVDF blots were blocked with Blocking Buffer (Beyotime, China) (1 h at 37 °C) followed by overnight incubation at 4 °C with the following rabbit polyclonal antibodies (against phosphorylated or total proteins): anti-rat p38 MAPK (1:500 dilution, Genetex, USA), anti-rat JNK (1:500 dilution, Genetex, USA), anti-rat ERK (1:300 dilution, Genetex, USA), anti-rat p65 NF-κB (1:500 dilution, Genetex, USA), or anti-rat GR (1:300 dilution, Genetex, USA). The blots were then washed four times for 5 min in PBST (Phosphate buffer solution containing 1‰ Tween-20) and incubated with horseradish peroxidase (HRP)-conjugated goat anti-rabbit IgG (Bioss, China) at 37 °C for 1 h, followed by four additional 5 min washes in PBST. The blots were developed with Chemiluminescent Assay Kit (ECL Star, Beyotime, China), analyzed with Digital Visualization Image System (Bio-Rad, USA), and band intensities were quantified with densitometry software (Image J, National Institutes of Health, Bethesda, MD). Band densities were normalized to β-actin, as follows: β-actin control (1:500 dilution, Beyotime, China) was detected after the removal of primary and secondary antibodies using Stripping Buffer (Beyotime, China).

### Inhibitor Administration

Immediately after CRBI, selective p38 MAPK blocker SB203580 (Sigma-Aldrich, USA, diluted in PBS) and JNK blocker SP600125 (Sigma-Aldrich, USA, diluted in polyethylene glycol) were administered subcutaneously, separately or jointly, each at 15 mg∙kg^−1^∙d^−1^ dose, for 7 days. [D-Lys3]-GHRP-6 (Phoenix Pharmaceuticals, Inc., Burlingame, CA, diluted in PBS), a specific blocker of GHS-R1a, was injected subcutaneously (5 mg∙kg^−1^∙d^−1^) at 6 h before each ghrelin infusion (200 nmol∙kg^−1^∙d^−1^), every day for 7 days. Polyclonal anti-rat TNF-α antibody (Janssen Pharmaceutica, Belgium, diluted in PBS) was i.p. administered (10 mg∙kg^−1^∙d^−1^, for 7 days). Four days after CRBI (or the initial inhibitor administration), rat peritoneal macrophages from each group (n = 3–5/group) were isolated for protein extraction, and serum samples (n = 5–7/group) were collected for TNF-α determination.

### Statistical Analysis

Unless stated otherwise, all continuous variables are expressed as mean ± standard error of the mean (SEM). Student *t* test and one-way ANOVA were used to calculate statistical differences between groups. Differences were considered as statistical significant if *P* < 0.05.

## Additional Information

**How to cite this article**: Liu, C. *et al.* Ghrelin accelerates wound healing through GHS-R1a-mediated MAPK-NF-κB/GR signaling pathways in combined radiation and burn injury in rats. *Sci. Rep.* **6**, 27499; doi: 10.1038/srep27499 (2016).

## Supplementary Material

Supplementary Information
