# Supplementary material for: Ghrelin accelerates wound healing through GHS-R1a-mediated MAPK-NF-κB/GR signaling pathways in combined radiation and burn injury in rats
Source: Sci Rep. 2016 Jun 7;6:27499. doi: 10.1038/srep27499 (PMC4895129; doi:10.1038/srep27499)
Supplement: Supplementary Information [file srep27499-s1.pdf]

Ghrelin accelerates wound healing through GHS-R1a-mediated MAPK-NF-κB/GR signaling pathways in combined radiation and burn injury in rats

Cong Liu[1], Jiawei Huang[1], Hong Li[1], Zhangyou Yang[1], Yiping Zeng[1], Jing Liu[1], Yuhui Hao[1,*], Rong Li[1,*]

Supplementary Figure S1

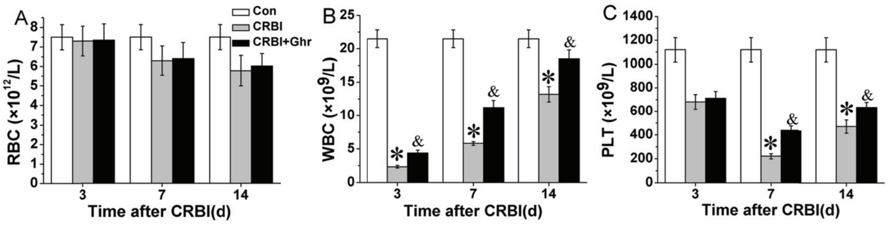

Supplementary Figure S1. Effects of ghrelin on hematopoietic function in CRBI rats. All CRBI rats received sterilize normal saline or ghrelin (200 nmol/kg) treatment subcutaneously for seven consecutive days, the count of RBC (A), WBC (B), and PLT (C) were detected at 3, 7, 14 days after injury. Data are presented as means ± SE (n=5-7) and compared by one-way ANOVA and Student-Newman-Keuls test: **P*<0.05 versus control group, $^{\&}P$<0.05 versus CRBI group.

Supplementary Figure S2

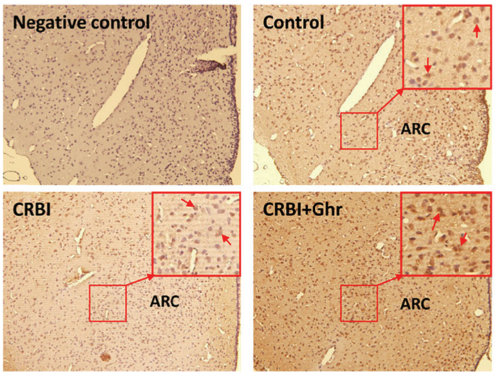

Supplementary Figure S2. Effects of ghrelin on GHS-R1a expression in the hypothalamus of CRBI rats. All CRBI rats received sterilize normal saline or ghrelin (200 nmol/kg) treatment subcutaneously for seven consecutive days. The hypothalamus of rats from each group (n=3-5) were harvested after the rats were infused and fixed with 4% paraformaldehyde at 7 days after CRBI, and then further fixed with 4% paraformaldehyde until assayed. The hypothalamus embedded with paraffin was sliced for immunohistochemistry (IHC) analysis. Rabbit polyclonal anti-rat GHS-R1a (1:300, Bioss, China) was applied to each slice and the levels of GHS-R1a in arcuate nucleus (ARC) were especially observed. GHS-R1a positive cells are marked with red arrows. Amplification: × 400.

Supplementary Figure S3

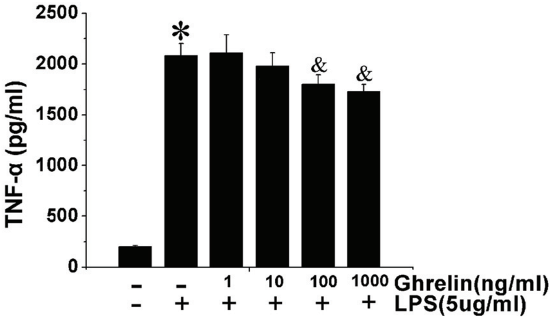

Supplementary Figure S3. Effects of ghrelin on TNF-α secretion of peritoneal macrophages irritated by γ-irradiation and lipopolysaccharide (LPS). Peritoneal macrophages isolated from

CRBI rats (or normal rats) at 4 days after injury were pretreated with saline or various doses of ghrelin (1, 10, 100, 1000 ng/mL) 4h before stimulated with 5μg/mL LPS. The TNF-α levels in liquid supernatant were detected by ELISA 24 h after stimulation. Data are presented as means ± SE (n=6) and compared by one-way ANOVA and Student-Newman-Keuls test: *P<0.05 versus control group; $^{\&}P$<0.05 versus single LPS treatment group.

Supplementary Figure S4

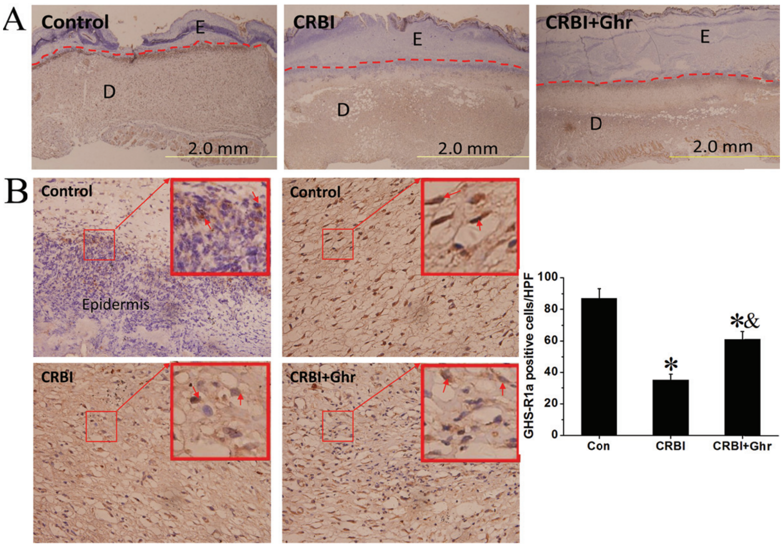

Supplementary Figure S4. The GHS-R1a expression in the rat skin during wound healing. At 7 days after injury, the granulation tissues of CRBI rats with or without ghrelin (200 nmol/kg) treatment were harvested. Otherwise, the granulation tissues in normal cutaneous wound healing (without CRBI injury) in rats were also isolated as a normal control. These granulation tissues (n=3-5/group) were fixed with 4% paraformaldehyde, and then embedded with paraffin until sliced for immunohistochemistry (IHC) analysis. Rabbit polyclonal anti-rat GHS-R1a (1:300, Bioss, China) was applied to each slice and the GHS-R1a levels in granulation tissues were especially observed. (A) The expression levels of GHS-R1a in epidermis and dermis of wound tissues (observation for cross-sections). D: dermis; E: epidermis. The junction between epidermis and dermis was marked with red dotted line. Amplification: ×40. (B) GHS-R1a expression in dermis (granulations) of wound tissues. GHS-R1a positive cells were marked with red arrows. The number of GHS-R1a positive cells in each HPF (High power field) from each group was counted and compared. Amplification: × 400. Representative results were given. Data are presented as means ± SE and compared by one-way ANOVA and Student-Newman-Keuls test: *P<0.05 versus control group, $^{\&}P$<0.05 versus CRBI group.

Supplementary Figure S5

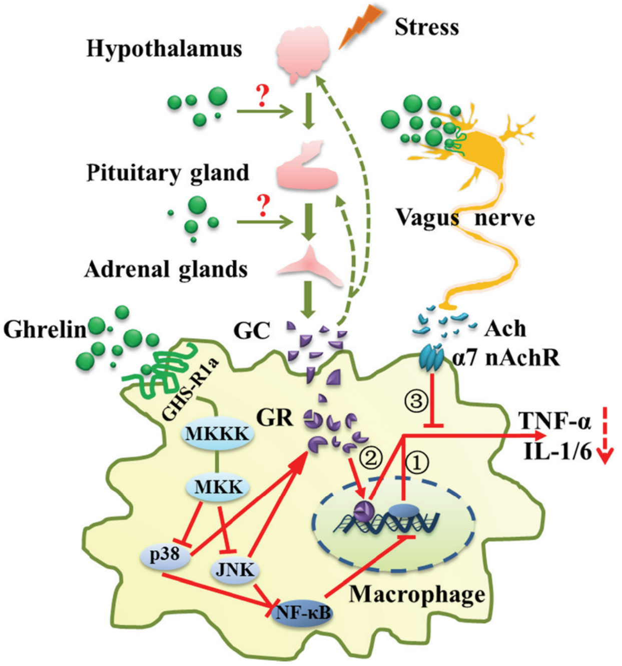

Supplementary Figure S5. The putative ghrelin-mediated anti-inflammatory mechanisms in macrophages. In this schematic diagram, ghrelin decreased the expression of proinflammatory mediators, such as TNF-α, IL-1, and IL-6 through three distinct pathways. ①Ghrelin inhibits the activation of p38 MAPK and JNK, and also the subsequent activation or translocation of NF-κB, through GHS-R1a, and finally alleviates inflammation. ②The inactivation of p38 MAPK and JNK increases the expression of GR, which enhances the interaction between GC and GR. The GC-GR complex binds to GRE, initiating the transcription of anti-inflammatory genes and inhibiting the transcription of proinflammatory genes. The effect of ghrelin on HPA is not clear in CRBI rats. ③Ghrelin activates the vagus nerve and enhances the release of Ach. Ach binds to α7 nAchR, influences the subsequent signaling pathways, and exerts an anti-inflammatory effect by interfering with the transcription and translation of proinflammatory genes. Abbreviations: p38 MAPK, p38 mitogen-activated protein kinase; JNK, c-Jun N-terminal kinase; NF-κB, nuclear factor kappa B; GHS-R1a, growth hormone secretagogue receptor 1a; GC, glucocorticoid; GR, glucocorticoid receptor; GREs, glucocorticoid response elements; HPA, hypothalamic pituitary-adrenal axis; Ach, acetylcholine; α7 nAchR, nicotinic acetylcholine receptors α7 subunit.
